# The influence of the Covid-19 pandemic and climate change on water use and supply: experience of Istanbul, Türkiye

**DOI:** 10.14324/111.444/ucloe.000061

**Published:** 2023-07-05

**Authors:** Ferhat Yilmaz, Dan Osborn, Michel Tsamados

**Affiliations:** 1 Department of Earth Sciences, University College London, Gower Street, London WC1E 6BT, UK

**Keywords:** water, Covid-19, climate, patterns of use, water management, adaptive management

## Abstract

The coronavirus (Covid-19) pandemic has affected not only populations around the world but also the environment and natural resources. Lockdowns and restricted lifestyles have had wide-ranging impacts on the environment (e.g., air quality in cities). Although hygiene and disinfection procedures and precautions are effective ways to protect people from Covid-19, they have significant consequences for water usage and resources especially given the increasing impacts of climate change on rainfall patterns, water use and resources. Climate change and public health issues may compound one another, and so we used a drivers, pressures, state, impact, response framework (not previously used to examine the actual and potential impacts of Covid-19 and climate change on water consumption and resources) to scope the main factors that may interact to affect water use and resources (in the form of reservoirs) using evidence from Istanbul, Türkiye, with some discussion of the comparative situation elsewhere. We modified initial views on the framework to account for the regional, city and community level experiences. We note that water consumption in Istanbul has been increasing over the last two decades (except in times of very low rainfall/drought); that there were increases in water consumption in the early stages of the Covid-19 pandemic; and, despite some increase in rainfall, water levels in reservoirs appeared to decrease during lockdowns (for a range of reasons). Through a new simple way of visualising the data, we also noted that a low resource capacity might be recurring every 6 or 7 years in Istanbul (a similar finding to Thames Reservoir in London). We made no attempt in this paper to quantify the relative contribution that climate change, population growth, etc., are making to water consumption and reservoir levels as we focused on looking at those social, environmental and economic factors that appear to play a role in potential water stress and on developing a drivers, pressures, state, impact, response framework for policy and adaptive management options for Istanbul and other large complex conurbations. If there are periodic water resource issues and temperatures rise as expected in climate projections with an accompanying increase in the duration of hot spells, the subsequent additional stress on water systems might make managing future public health emergencies, such as a pandemic, even more difficult.

## Introduction

Water is a fundamental part of life in any society, and vital for people’s health and well-being, agriculture, businesses and the environment. Water use patterns change on a daily, seasonal and annual basis. Resource levels and water use in different parts of any country can be affected by many factors: for example, normal variations in rainfall that affect river flows (that might be used for abstraction); changes in reservoir and aquifer levels due to recharging or drawdown; and changes in the amount of irrigation needed for commercial crops or the maintenance of private and public gardens during periods of hot weather.

Water management must ensure that water remains at sufficient levels to sustain the environment in the long term to guarantee future generations’ prosperity. Maintaining water needs for drinking, industrial, agricultural and other usages are expected to be one of the most important challenges for societies, as is already apparent in some parts of the world [1]. Enhanced vulnerabilities from climate change, such as those that might arise from more intense and frequent storms, heatwaves and sea-level rise, could increase water-related stresses on society, the economy (including food production) and the environment [2].

Climate change puts established patterns of resource management and usage at risk because of long running and often gradual trends in overall temperatures and rainfall patterns and changes in the frequency or intensity of extreme events such as flooding and drought [3]. Managing these changes can be more difficult if other trends, such as those in population levels or demographics, need to be incorporated into planning. Public health emergencies, such as pandemics, may complicate matters further.

The coronavirus (Covid-19 pandemic)^1^ is an extreme event of a particular kind: a global health emergency that might affect water usage patterns or wastewater management because of public health concerns, if nothing else, and, thereby, available water resources over a period of at least one or two years. This period would be longer if the pandemic could not be controlled and/or it led to long-term shifts in water use, as might be the case if, say, working and travel patterns changed permanently. In such circumstances, the impact on water consumption and thereby the resilience of resources might be difficult to predict and quantify.

At the outset, we thought it likely that the pandemic would increase water use because of the need for additional public health measures involving extra cleaning (e.g., of people, public buildings and transport systems). This could be taken as the starting hypothesis for the study. Also, we wanted to investigate whether, if a pandemic occurred in the future, when climate change had led to further alterations in weather and hydrological conditions than those already apparent (e.g., more frequent or more severe droughts), that would stress Istanbul’s water system further. This was a more complex idea to test because the impact of such compound risks on a system are difficult to anticipate. Thus, to account for these complexities and to be able to consider other relevant factors that might emerge in our study we investigated the use of a drivers, pressures, state, impact, response (DPSIR) framework approach in addition to considering hypothesis testing [4].

While previous studies have used the DPSIR approach to analyse water-related problems and identify the factors involved [5,6], there has been no previous use of a DPSIR framework to examine the actual and potential impacts of Covid-19 and climate change on water consumption and resources. Our initial DPSIR framework shows climate and pandemic factors that, at the outset, we believed could affect water resources, water consumption and the supply system of Istanbul, Turkiye, some of which are set out in Yilmaz et al. [7]. We then looked for evidence of the influence of such factors on water resources and water consumption in Istanbul now and in the future. We did this with the help of climate change tools and scenarios because climate change makes extreme events such as droughts more likely and such events could make a future pandemic more difficult to manage. We hoped that by gathering evidence from a range of sources, we would be able to modify the initial DPSIR diagram so that it could provide a framework for thinking through what kind of influences on water supply and consumption patterns were required to be incorporated into future adaptive water management planning for this, and other, major urban centres. The water system of a large municipality might then be more resilient because it had been designed in a manner that provided for adaptation to trends in climate change impacts, and to changes in frequency and severity of extreme events of different kinds. Adaptation would include making infrastructure more resilient and making preparations to affect human behaviour towards water resources.

Examining the situation in Istanbul through a DPSIR lens was helpful to our wider studies because we are interested in identifying the factors that influence the level of risks that need to be managed in different municipalities in Türkiye and elsewhere.

In this paper, for Istanbul, we examined data and information on how the pandemic, water usage and availability changed, during the Covid-19 pandemic.

We focused on events in 2020 and compared these with situations in recent years, both during the pre-pandemic period (2017–2019) and the pandemic period (2020–2022). We also used data from previous times to provide a context for these studies of recent years (since 1937 for rainfall and temperature anomalies, since 2000 for water consumption and since 2005 for water reservoir levels). The period studied, which is the early stage of the Covid-19 pandemic in 2020, is significant as it represents a unique time where the daily activities of people and industries were drastically altered due to the restrictions and lockdown measures implemented by the government to curb the spread of the virus. This period provided a rare opportunity to observe and analyse the changes in water use and resources in a large complex urban system (Istanbul), which could provide valuable insights for future policy and management options under global warming and other potential shocks and stresses. We used Istanbul as a case study of compound risk as this is Turkiye’s largest centre of population and because Istanbul experienced drought conditions in the recent past in the period around 2007. We quantified the changes in water use and water reserves during the period in which Türkiye went into and emerged from the initial Covid-19 lockdown state in 2020. When people were advised to stay at home as much as possible, the demand for water might be expected to increase in domestic settings and decrease in many business settings and schools, for example. We also expected changes in patterns of water use that could have consequences for water supply for domestic use due to infrastructure failure or capacity limitations. The impact any of this would have on overall water consumption was uncertain at the beginning of the study.

## Approach and methodology

### Study area and environmental conditions prevailing in Istanbul

**Figure 1:**
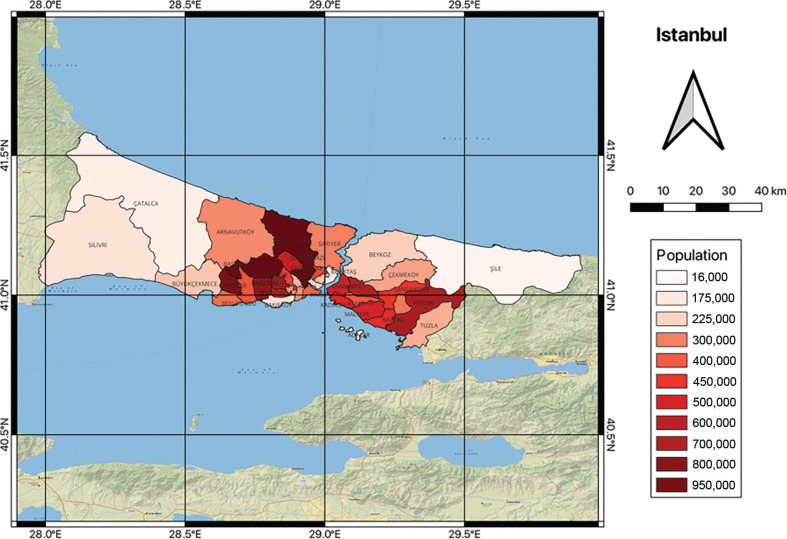
Istanbul Municipality area showing district populations in 2020 [8].

In this study, Istanbul (5343 km^2^), the most populous city in Türkiye (over 15 million people) (Fig. 1), has been considered in detail. Istanbul is largely reservoir-dependent, there being no substantial groundwater sources that have yet to be exploited.

**Figure 2:**
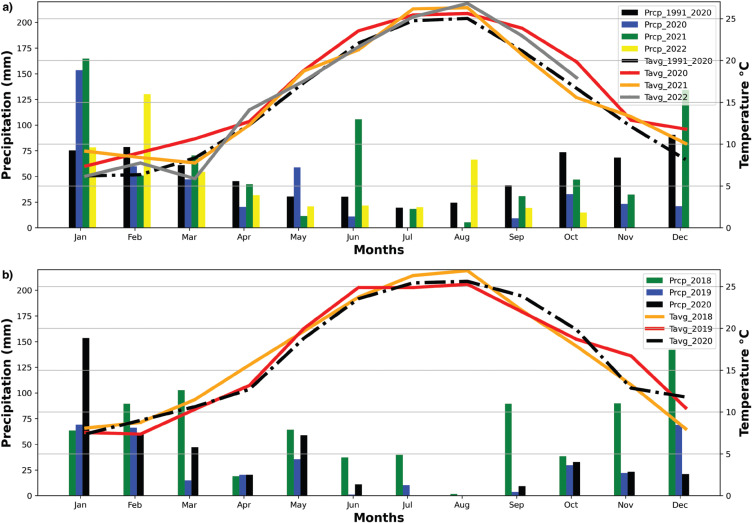
Monthly temperature (°C) and total precipitation (mm) in Istanbul. (a) average monthly temperature and precipitation in Istanbul, 1991–2020, compared with the same data for the pandemic years 2020–part of 2022; (b) detailed information for 2018, 2019 and 2020.

Assessing the impact of the pandemic on water resources needs to include consideration of the prevailing environmental conditions relevant to the hydrological cycle. Figure 2 shows (a) the average monthly temperature and precipitation in Istanbul over the period 1991–2020 in comparison to the same data for the pandemic years 2020–part of 2022 and (b) detailed information for 2018, 2019 and 2020. In 2020, the first quarter (January, February and March) were the wettest months and there was almost no rainfall in the summer months (June, July, August and September), which are also the hottest times of the year. The average temperatures of the last three years were close to or higher than the 30-year average (1991–2020).

## Initial DPSIR framework

**Figure 3:**
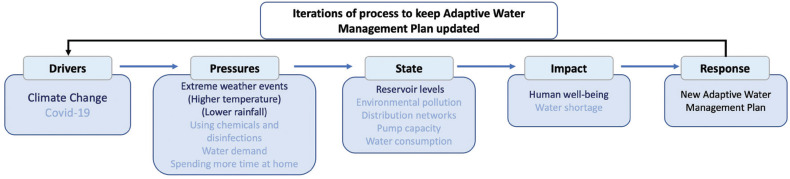
DPSIR framework: overview of factors likely to be involved in changing water consumption in pandemic circumstances that might lead to supply challenges (light blue text) with the equivalent climate change factors (dark blue text).

In this study, a DPSIR framework was set out in two ways (an initial view and an expanded version based on the findings of this study). Smeets and Weterings [4] detail the origins of the DPSIR approach introduced by the European Environment Agency. The initial framework for Istanbul (Fig. 3) includes the factors thought most likely to be involved in changing water consumption in pandemic circumstances (light blue text) together with the equivalent climate change factors (bold blue text) that might be important in the longer term or in the event of a climate-related extreme event coinciding with a pandemic. As noted, climate change and Covid-19 are the main drivers of the system, and the pressures exerted by climate change and the pandemic include extreme weather events, using chemicals and disinfections, water demand and spending more time at home during the pandemic. The state in response to the pressures is characterised by changes in reservoir levels, environmental pollution, distribution networks and pump capacity and increased water consumption. The impacts of the current state include human well-being and water shortage, which will be addressed by a new adaptive water management plan (response, the measures taken by policymakers and other stakeholders).

Pandemic and climate factors co-located in the sectors of the model (Fig. 3) might amount to interacting factors or compound risks in their own right. We assume that governmental bodies and others would want to respond to issues by developing an adaptive management plan to deal with at least the climate change aspects of the hazards and risks and adjust their emergency planning to account for impacts on pandemic preparedness and response.

### Data rationale

We used data and information from three time periods to obtain a wider context for the events of the pandemic, an understanding of the pandemic’s consequences, and to gain insight into what it might all might mean for the long-term management of water in Istanbul. To capture the topical events of the pandemic we have used a mixed methods approach using both quantitative as well as qualitative data.

Information on water and average weather conditions (precipitation, temperature) was gathered for recent decades with time periods limited by data availability and consistency. Reference periods were selected as appropriate to each dataset. The periods were from 2000 to the early part of 2022 for water and the late 1930s to the present day for the weather data. This information provided both a context for the water-relevant events of the pandemic and a foundation for examining those aspects of climatic trends that might make future pandemics more difficult to manage if, say, they occurred coincidentally under drought conditions. This data allowed us to examine water use and water resource status monthly for each of the years from 2000 and examine, using annual averages, trends and variability in key aspects of the weather patterns that, when accumulated, indicated the climatic trends and tendencies in Istanbul for these decades from 1930 to the present day.

For examining events linked to the years immediately preceding the pandemic we used similar data types to that used for the analysis of recent decades plus information of other kinds to gain an understanding of how the pandemic affected the people of Istanbul socially and what the consequences were for the water system of measures aimed at managing the pandemic.

Because data from recent decades indicated climatic trends were in play in Istanbul that were likely to make any vulnerability in the Istanbul water system worse, we also examined, using tools based on climate change modelling, how temperature (which might drive higher water use), the frequency of exceptionally warm periods (which might drive drought conditions) and precipitation might vary in Istanbul in the future for selected decadal periods going from the present time to the end of the 21st century. This provided additional information that could help develop thinking about how to manage water in Istanbul in the future.

### Data sources

#### Weather data

Temperature and rainfall records were retrieved from the National Oceanic and Atmospheric Administration and the Turkish Met Office [9,10].

#### Water consumption

Istanbul water consumption and reservoir level data were obtained from the Istanbul Municipality [11–13].

#### Population

Data in Türkiye is published by the Turkish Statistical Institute each year [8]. We used data for the years for which we had information on water use.

#### Climate change

Climate projection data (temperature, rainfall and warm spell duration index) is presented on the World Bank’s Climate Change Knowledge Portal (CCKP), including the Coupled Model Intercomparison Project (CMIP) Phase 5 and Phase 6 collections, which serve as a repository of model runs with consistent emission scenarios to standardise some of the output generated by diverse modelling groups. As the spatial scale of a global climate model’s output is too large to characterise climate over small and specific areas, downscaling to a finer resolution is necessary. Thus, statistically downscaled climate variables over the studied region were downloaded from the World Bank’s CCKP [14]. We chose RCP 2.6 as a low emission scenario (one where the Paris Agreement was met), RCP 4.5 as an intermediate emission scenario, RCP 6.0 as a stabilising emission scenario and the higher RCP 8.5 scenario if the Paris Agreement was not successful at reducing global emissions. We selected four different periods to examine (2020–2039, 2040–2059, 2060–2079 and 2080–2099). In order to look at the specific location of Istanbul, the outcomes of CIMP5/6 ensemble model for RCP 2.6, RCP 4.5, RCP6.0 and RCP 8.5 were extracted [14]. We used the Portal as it is one that policymakers and government agencies can get ready access to and might use for planning adaptations to climate change.

#### International comparisons

As part of our international comparisons (see below) we used the National Hydrological Monitoring Programme’s Monthly Hydrological Summaries, including water reservoir levels across the UK [15].

#### Air quality and traffic movements

Additional analysis of air quality and traffic movements was used to support the widely held contention that the Covid-19 pandemic would lead to changes human behaviour in Istanbul (and other major cities). All these data sources/sets can be freely accessed from the link provided in the references [16] at https://doi.org/10.5522/04/19122179.

#### Information from the government and the media

We used information gathered from the news and social media as well as information issued by local government bodies or published in authoritative reports (e.g., from water utilities and consultancies) as well as material in the academic literature. We used this range of sources to try to capture the fast-changing circumstances of the pandemic. To increase the likelihood that our findings might be generalised, we drew on information from other countries, including the UK, the United States, Germany, Italy and Brazil.

### Data visualisation and analyses

**Figure 4:**
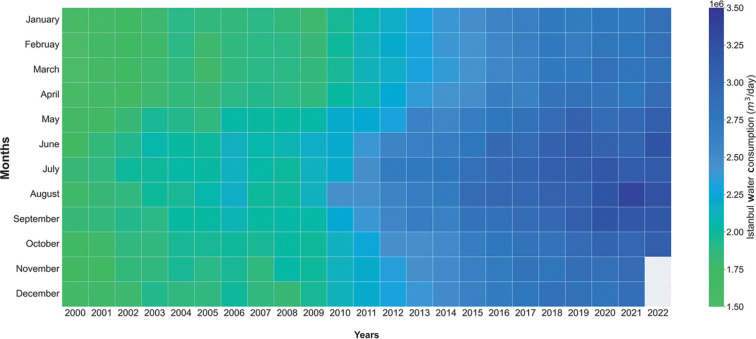
Monthly water consumption (million m^3^/day) in Istanbul from January 2000 to October 2022.

**Figure 5:**
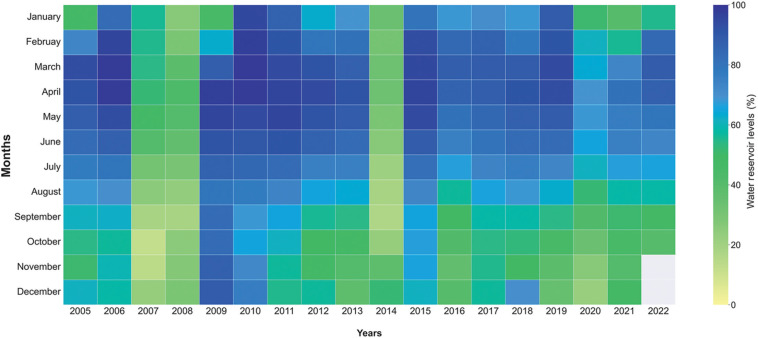
Monthly water reservoir levels (as % of capacity) in Istanbul from January 2005 to October 2022.

**Figure 6:**
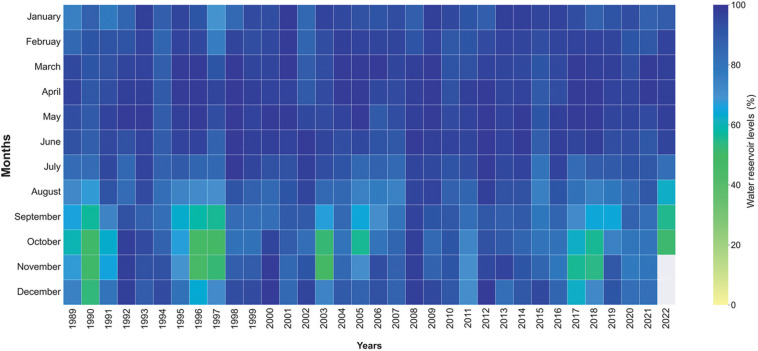
Monthly water reservoir levels (% live capacity) in London reservoirs from January 1989 to October 2022 [17].

Visual representation of data or information might make it easier to understand and identify any time-related changes in large data sets, so water consumption and reservoir data have been visualised as in Figs 4–6. For visualisation, we used Matplotlib, a Python plotting library that generates and customises various types of plots, and Seaborn, a plotting package that builds on top of a Matplotlib library [18].

## Results

### Main case study: water use and resources in Istanbul

First, we set out information on water use in Istanbul that is necessary to provide the basic context for examining the way the DPSIR approach might be used to better understand the impact that the two DPSIR drivers, Covid-19 and climate change, might have on the water system of Istanbul. We then provide a range of information on human activity in Istanbul during the early phases of the pandemic that necessarily draw on some information from other places in Türkiye. This further contextualises the study and allows more nuanced interpretation of monthly water use changes across Istanbul in the years after the beginning of the pandemic as, together with the human activity information, these indicate the complexity of pressures on water systems that a pandemic can bring. We then examine the state of the water system via consideration of reservoir capacities as this will be a main factor affecting water supply and use and is known to be central to the Istanbul water system from earlier extreme events such as the drought of 2007/2008. This in turn led to consideration of the short- and longer-term climatic factors that might influence the state of the water system and cause additional pressures – such as when higher temperatures in the summer months might cause an increase in use. Following on from that we examine how the climate change might increase pressures still further in the future and make another pandemic even more difficult to manage. To ensure the Istanbul case study was not exceptional in international terms we compared Istanbul with other studies of national, regional or urban water systems during the Covid-19 pandemic to develop a more sophisticated view of the many interactions that needed to be taken into account to build resilience. This includes using some indications of the impacts of the pandemic on the pattern of water use or potential pollution of water courses. Impacts and responses are considered in the Discussion section, where a modified DPSIR framework is set out that might help build resilience in water systems in Istanbul and elsewhere.

#### Water use context for the early pandemic period

**Figure 7:**
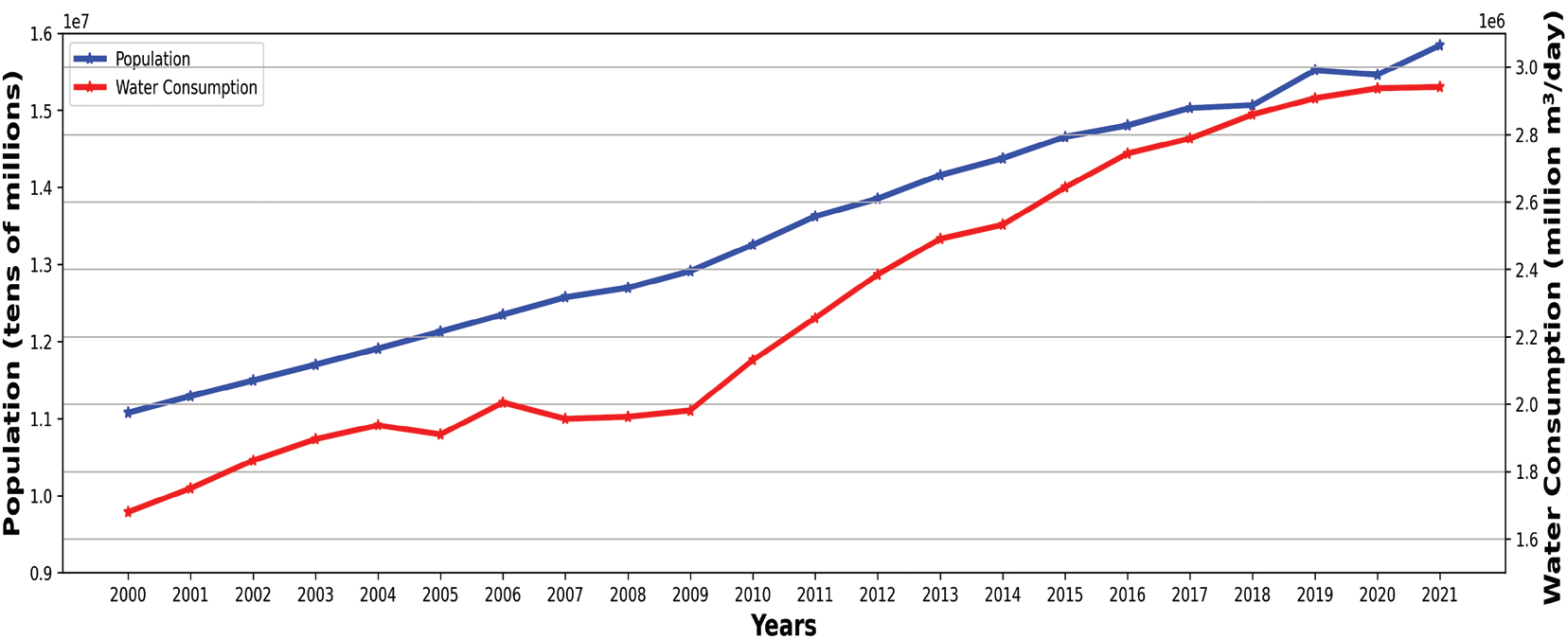
The comparison of population and water consumption in Istanbul from 2000 to 2021.

Figure 4 shows that water use in Istanbul has varied month by month from 2000 to 2022. Water consumption in Istanbul has nearly doubled. It has gradually increased since the 2000s (as shown by the shift from green to blue and darker blue) from an average of 1.6 million m^3^/day to 3.1 million m^3^/day. Water use was consistently higher in the summer. The overall increase in water use may be attributed to population increase, industrialisation and rising standards of living and/or lifestyle choices (such as leisure uses) that go alongside increasing consumption levels in general. Additionally, Fig. 7 shows that over the 21 years the population increased by about 40%, while water consumption rose by 70%.

**Figure 8:**
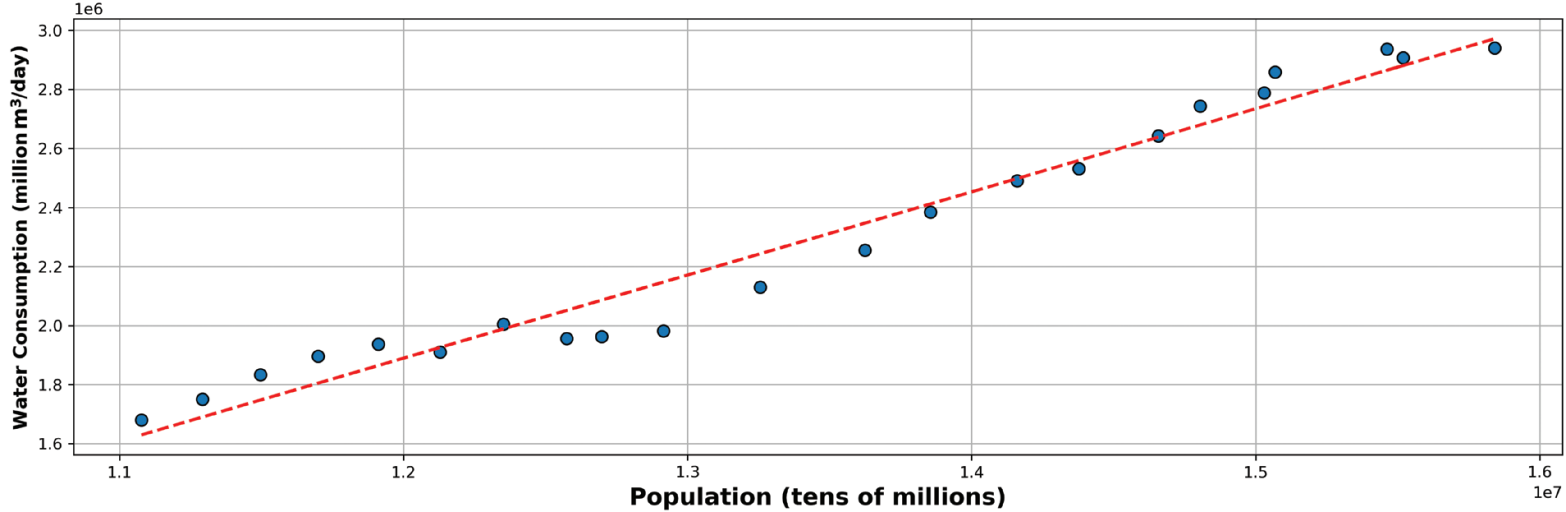
The relationship between population and water consumption (m^3^/day) from 2000 to 2021 in Istanbul. Overall, in this period, water consumption per head of population moved from about 150 L/day to 190 L/day, increasing by over 25%.

Although population and water consumption (Fig. 8) both increase over time (and therefore show a strong Pearson correlation of r = 0.98, n = 20, *P* < 0.001), there have been periods when water consumption grew very little, for example, between 2005 and 2007–2009. This can be linked to low rainfall and drought periods in 2005 and 2007 [19], with people and businesses adjusting their behaviour to reduce consumption following communications from governmental bodies and the media about water shortages.

#### Water use and changes to human activities in the Istanbul area during the early stages of the Covid-19 pandemic

As has been recorded for many major cities (see below in the section on international comparisons), there were marked impacts on human activity in the early stages of the pandemic due to lockdown restrictions.

In order to obtain some separate evidence (other than that on water resources and consumption) that the pandemic restrictions affected human activity in Istanbul, we examined air quality parameters, and these suggest that air pollution in Istanbul was lower in 2020 than in 2019, indicating decreased human activity consistent with Covid-19 restrictions. Air quality parameters such as particulate matter (PM)10, sulfur dioxide (SO_2_) and carbon monoxide (CO) provide good evidence of these changes [12]. Annex–I in [16] shows how the levels of these parameters decreased in Istanbul. After confirmation of the first cases in Türkiye in March, air quality improved as pollutant levels fell in late March when national restrictions (working remotely, closing restaurants and full lockdowns) were announced and applied. This indicates less commercial activity overall.

**Figure 9:**
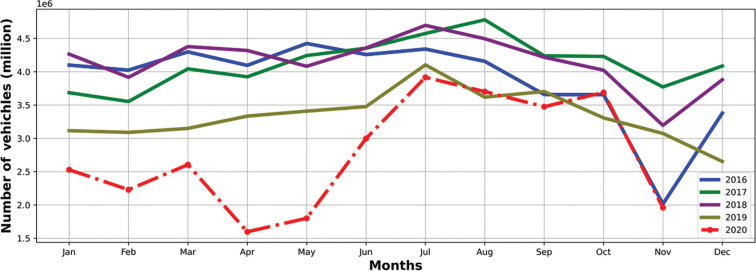
Monthly total number of vehicles at Kadikoy, Istanbul Metro [13]. It is clear that the total number of vehicles measured on one of the main roads in Istanbul decreased after many governmental restrictions (e.g., full lockdowns) were applied from early April 2020. This suggests that traffic was less during the pandemic even if people may be ordering products and services online, delivery movements increased. It does affect the total number of vehicles in an increasing way during the first pandemic period. The effect persists throughout the pandemic period matching only the lower values for previous years in autumn 2020.

In addition to air quality parameters, Fig. 9 shows how monthly sensor-based numbers of vehicles at Kadikoy, Istanbul Metro, have changed since 2016. The station (near the Optimum Shopping Centre) is located on one of the main connections from Asia to Europe through the Eurasia Tunnel. As the road is actively and intensively used during the day, vehicle movements could be an indicator of how Covid-19 restrictions affected transportation in Istanbul. There was a sharp decrease in April and May 2020, during the national Covid-19 restrictions (working remotely, closing restaurants and full lockdowns).

Data underpinning the visualisation in Fig. 4 show that in March 2020, when Covid-19 started to spread in Türkiye, water consumption was higher than in March 2019 (from 2.73 million to 2.85 million m^3^/day). This increase of about 120,000 m^3^/day (or about 3%) could reflect additional water use due to the increased emphasis on hygiene measures such as hand washing or street disinfection (although it is uncertain if this later increase is included within the figures). This might appear to be a slight increase. However, in March and April, when the Turkish government decided to move to a lockdown in big cities, including Istanbul, substantial numbers of people decided not to stay in a highly populated city and went to their hometowns or summer houses. The movement of people seemed substantial such that the Mayor of Bodrum, a coastal town in the Muğla area, stated ‘*Please do not come to Bodrum; Bodrum is full*’. Many vehicles entered Bodrum that were registered in Istanbul or Ankara [20]. The first Covid-19 case in Bodrum was confirmed in a person coming from Istanbul before the start of travel restrictions among the big cities in Türkiye [21]. These temporary internal migrations probably explain why consumption in Istanbul fell in April by an average of 42,210 m^3^/day when traffic movements were also at a minimum.

**Figure 10:**
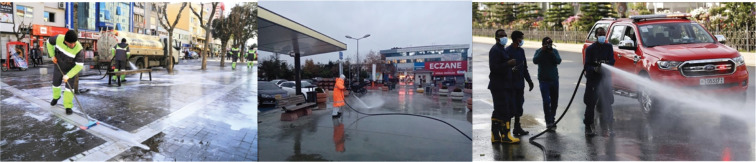
Street disinfection photos from Türkiye [Usak (left) and Istanbul (middle)] and Ethiopia (right) during the Covid-19 pandemic [22–24]. In addition to street photos, the Municipality of Bolu, a small city in Türkiye, posted a video on its official social media account (Instagram): https://www.instagram.com/p/CIao9VJJJC0/?igshid=1irbte6t7xd5j with the caption: ‘You are at home, we are on duty, wish everyone good health’ on 5 December 2020. Although Bolu was exceptional and extremely dry according to the Standardized Precipitation Index (SPI) from October to December 2020 [25], the municipality used lots of water and chemicals to clean its main streets.

From June, when Covid-19 cases started to decrease in Türkiye as a whole, some people began returning to Istanbul, and water consumption gradually increased by 141,609 m^3^/day to the end of the summer in August. Continuing public health measures such as an increased emphasis on hand washing and the sanitisation of public spaces with water-based disinfectants (Fig. 10) could all be expected to lead to increased water consumption in comparison to use in recent years.

#### Monthly water use per head

**Figure 11:**
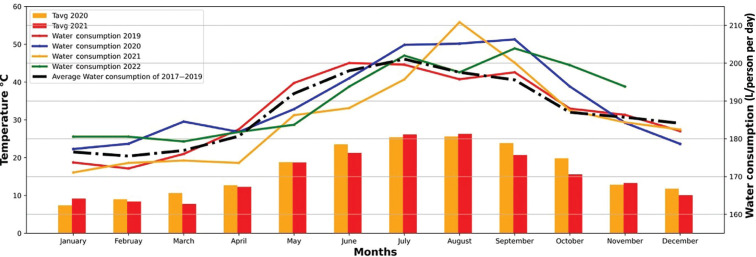
Water consumption (L/person per day) for 2019–2022 (years shown individually) and the average pre-pandemic consumption (2017–2019) together with 2020/21 monthly temperature average in Istanbul. During the pandemic period, daily water consumption per head was above both the average and range of consumption in the previous three years and did not fall back as temperatures fell as it had done in previous years, suggesting that the higher consumption of water seen for July–September was due to the pandemic and not to other factors (such as an extended period of high summer temperatures).

Expressing the water use data in terms of consumption per head per day, as in Fig. 11, creates further insights, especially when the first Covid-19 pandemic year of 2020 is compared to the same data for the three years preceding the pandemic (data from earlier years was not used to avoid complications linked to the rising water use rates in Istanbul).

Water use in 2020 (blue line) was higher than the average of 2017–2019 (black dashed line) for several months between March and October (an extra 2.56 L per person per day was consumed across the year as a whole, assuming a constant population for Istanbul although we know this was not the case due to the scale of temporary migrations). A similar effect was found in other countries as well (see Discussion). In contrast, during the period people left Istanbul temporarily (April and May 2020), water use was lower than the 2017–2019 average – for instance, in April, it was 2.73 L/person per day lower than in March. As people gradually returned to Istanbul from the second quarter of 2020, there was a steady increase in water consumption, with consumption in July–October higher than the 2017–2019 average. In the pandemic year of 2020, along with rising summer temperatures, which generally cause additional water usage, water consumption continued to climb above the three-year average right through until September, when, usually, consumption would have fallen back. This suggests that the Covid-19 pandemic placed an additional exertion on Istanbul’s water resources (about 2% greater across the year as a whole assuming a constant population). Perhaps the most striking finding was that in 2020 water consumption per head in Istanbul did not decline in July, August and September as it had in earlier years. The difference in this period between 2020 consumption and the three-year average was about 10 L/person per day, equivalent to approximately 150,000,000 L/day (or 150,000 m^3^/per day). An additional complexity of interpretation is that in the last three years, religious holidays (for Islam) were in August and September. This might have an impact on water consumption as some people went to their hometown during the religious holiday.

#### Water resources as a measure of system state

**Figure 12:**
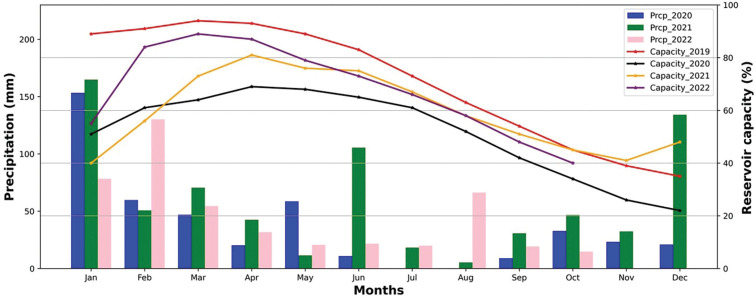
Comparison of water reservoirs’ live capacity (%) and total monthly precipitation (mm) in Istanbul. Live capacity in 2020 did not reach 2019 levels and was already close to the minimum level for 2019 (December) in August. Water resources were not replenished to 2019 levels in 2020 despite higher rainfall in early 2020 than in 2019. This indicates higher water use, some of which, say for the cleaning of public spaces, might not have been recorded in the standard figures given their emergency nature.

Yilmaz et al. [7] provide information on the water resources of Istanbul, which are largely reservoir-based. Periodically, reservoir levels are low, suggesting that Istanbul is close to experiencing drought conditions should rainfall be lower than usual. Monthly water reservoir levels (as % of live capacity) from 2005 are given in Fig. 5. In most years, reservoirs are at their highest levels in late-winter and spring months and lowest in the autumn because of the increased consumption during the summer and lower rainfall together lead to resource depletion. Reservoir levels were at their lowest level in this record in 2007, 2008 and 2014. These years are shown clearly in the visualisation of Fig. 5. In the pandemic year of 2020, the highest reservoir level – a nevertheless relatively low 69% was observed in April, when human activity was at its lowest and public health measures were at their maximum extent. Normally, an increase in reservoir levels is expected with increased rainfall through the end of the year. However, the decreases in reservoir capacity that started in the spring months of 2011, 2013, 2019 and 2020 saw limited recovery (Fig. 12). In the last two years (2019–2020), capacity did not recover throughout the year, so even without the impact of Covid-19, there was a case to be made for taking a precautionary approach and encouraging water saving. There are indications in this limited dataset that, effectively, near-drought conditions impacting Istanbul may occur every 6–7 years. Within the data for our limited time period: a severe drought occurred in 2007/8, and not dissimilar conditions occurred in 2014/15, and late in 2019 and 2020 and perhaps into 2021.

## Possible influence of changing climatic conditions

**Figure 13:**
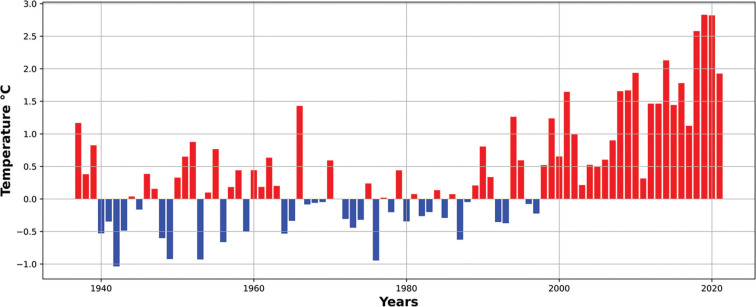
Istanbul temperature °C anomaly. This figure plots the average temperature anomaly data from 1937 to 2021 relative to 1961 to 1990 values at the Florya Metro Station, Istanbul. There is an increasing trend in the anomaly in Istanbul, especially after the 1990s. Since then, some of the relatively larger positive anomalies have occurred every 6–7 years (1990/1, 1995/6, 2000/1, 2007/8 and 2014/5), indicating that low reservoir levels occur when temperatures are anomalously high.

**Figure 14:**
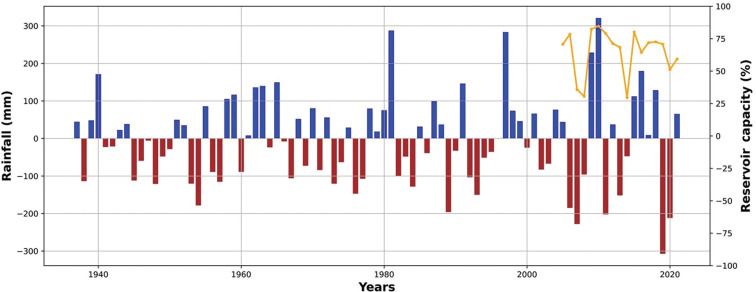
Istanbul rainfall (mm) anomaly with changes in capacity (%). This figure plots the total rainfall anomaly data from 1937 to 2021 relative to 1961 to 1990 values at the Florya Metro Station, Istanbul. Since the 1970s, the rainfall anomaly has changed sign often. There may be some evidence of a cycle in rainfall anomalies as well (1990/1, 1994/5, 2001/2, 2007/8, 2014/5 and 2019/20). This suggests that low reservoir levels occur when there is a lower rainfall (see the relationship between rainfall and capacity levels after 2005).

Figure 2 provided a context for the pandemic years of 2020 and 2021 in terms of the prevailing temperatures in Istanbul compared to a 1991–2000 baseline. The data suggest that whilst temperatures were similar or higher in the pandemic years, rainfall was more variable, For example, June 2021 was much wetter than the average and some autumn months were much drier. Such short-term variations in temperature and rainfall are notoriously difficult to interpret and this adds to the challenges associated with managing water resources. Thus, we examined a longer run of temperature and rainfall data for Istanbul to see whether, over a number of decades, there was any indication that temperature was increasing (this might lead to increased water consumption) or rainfall becoming more variable (this might lead to drought conditions and would increase the uncertainty in managing resources to meet demand). Figure 13 (temperature anomalies) shows clearly that the temperatures in Istanbul have been rising steadily since about 2000. There is also a suggestion in the data that there may be a similar periodicity in higher temperature anomalies and low water resource levels. Data in Fig. 14 (rainfall anomalies) suggests that rainfall patterns are also becoming more variable and again there is some suggestion that periods of low rainfall coincide with low reservoir levels. Taken together this suggests that as well as the additional water demands of the pandemic an influence of climate change may also be evident. As this has implications for water resources in the future, we examined whether these trends in temperature and tendencies in rainfall patterns were likely to continue into the future using climate modelling tools readily available to managers of water resources in many countries.

### Implications for future water consumption and resources under climate change

#### Projected temperature and rainfall

Given the evidence above (in Figs 5,12–14) that Istanbul may be subject to rising temperatures and variable rainfall and thus drought-like conditions relatively frequently, we examined four climate change emission scenarios (RCP 2.6, RCP 4.5, RCP 6.0 and RCP 8.5) at four different time periods to better understand whether conditions would be right in the future for such events to become more frequent or more intense. Projected temperature and rainfall patterns compared to the reference period (1986–2005) have been placed in the University College London (UCL) Research Data Repository [16].

Overall, the climate projections given in Table 1 suggest that temperatures will rise in Istanbul, but rainfall will not increase. Rainfall may decline most under those scenarios where temperatures move to potentially very high values (the mid- or even high-40s°C). With current pressures and limited water resources, these locally expected trends in temperatures and rainfall may have significant impacts on water availability over the upcoming decades especially if there is lower rainfall at times of the year when reservoirs are currently replenished. Given the long-term implications for increased water use in Istanbul as a result of higher temperatures and given that the population might increase further, dealing with the public health aspects of a future pandemic might indeed become even more challenging, especially if it occurred in a drought year. Much may depend on how long warm and/or dry spells last in the future. It is appreciated that the argument here is complex and depends on a range of social technological and economic issues, but it would seem wisest to plan for more water use to cope with a pandemic-like public health emergency.

**Table 1.** Temperature and rainfall changes

Scenarios	Parameters and future time periods against a baseline of 1986–2005							
	Temperature				Rainfall			
	2020–2039	2040–2059	2060–2079	2080–2099	2020–2039	2040–2059	2060–2079	2080–2099
RCP 2.6 (Low emission scenario)	Between 1 and 2°C per month increase is expected for all the months, with potential increase up to 3.2°C, in August	Between 1 and 2.5°C per month increase is expected for all the months, with potential increase up to 3.5°C, in August	Between 1 and 2.5°C per month increase is expected for all the months, with potential increase up to 3.7°C, in August	Between 1 and 2.5°C per month increase is expected for all the months, with potential increase up to 3.6°C, in August	Same as the baseline values in all months with potential changes up to ± 30 mm	Changes up to ± 40 mm	Changes up to ± 40 mm	Changes up to ± 35 mm
RCP 4.5 (Intermediate emission scenario)	Between 1 and 2°C per month increase is expected for all the months, with potential increase up to 3.5°C, in July	Between 1 and 3°C per month increase is expected for all the months, with potential increase up to 4.2°C, in July	Between 1.5 and 3.5°C per month increase is expected for all the months, with potential increase up to 5.1°C, in July.	Between 2 and 4°C per month increase is expected for all the months, with potential increase up to 5.5°C, in July	Same as the baseline values in all months with potential changes up to ± 30 mm	Changes up to ± 40 mm	Changes up to ± 40 mm	Changes up to ± 35 mm
RCP 6.0 (Stabilising emission scenario)	Between 0.5 and 1.5°C per month increase is expected for all the months, with potential increase up to 2.7°C, in August	Between 1 and 2°C per month increase is expected for all the months, with potential increase up to 3.5°C, in August	Between 1.8 and 3.8°C per month increase is expected for all the months, with potential increase up to 4.8°C, in August	Between 2.5 and 4.2°C per month increase is expected for all the months, with potential increase up to 5.3°C, in August	Same as the baseline values in all months with potential changes up to ± 30 mm	Changes up to ± 40 mm	Changes up to ± 40 mm	Changes up to ± 35 mm
RCP 8.5 (High emission scenario)	Between 1 and 2°C per month increase is expected for all the months, with potential increase up to 4.2°C, in July	Between 2 and 3.5°C per month increase is expected for all the months, with potential increase up to 5.4°C, in July	Between 3 and 4.5°C per month increase is expected for all the months, with potential increase up to 7°C, in July	Between 4 and 6°C per month increase is expected for all the months, with potential increase up to 9.2°C, in July	Lower than the baseline values (−10 mm) in all months with significant changes up to −35 mm	Changes up to −35 mm	Changes up to −40 mm	Changes up to −45 mm

#### Projections of future warm spells

**Figure 15:**
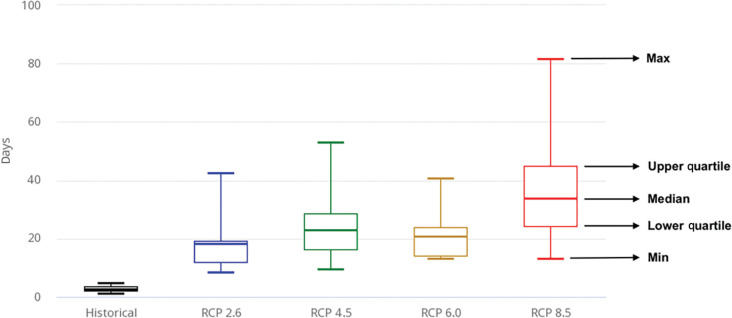
Warm Spell Duration Index in Turkiye for the period 2040–2059, the only time period available in [14], for the reference period 1986–2005.

Because it appeared that temperatures in Istanbul would rise substantially in the future under some scenarios we thought that the Warm Spell Duration Index (WSDI), defined as the count of days with at least six consecutive days when daily Tmax > 90th percentile of the reference period (1986–2005) [26], would be a useful indicator of periods when water use might be high or conditions were favourable to drought. Figure 15 shows the historical Türkiye WSDI for 1986–2005 and the projected WSDI by 2060 under four emission scenarios. Even in a low emission scenario (RCP 2.6), warmer periods seem likely to last about nine times longer than in the reference period (1986–2005). This will likely place more pressure on water resources, as people under high temperature conditions tend to use more water for recreational and other activities.

### International context to observations from the case study in Istanbul

Before modifying the original DPSIR framework (Fig. 3), we wanted to establish whether the Istanbul situation was unique or rather was similar to that found in other major cities or regions of countries during the pandemic, so we searched for comparative information in academic publications, reports from governments and companies and in the media.

#### Water use

Limited comparisons are possible between Istanbul and other areas at present due to a lack of comparative data for the pandemic period. This is due to natural–environmental variables, such as varying climatic patterns, as well as variations in governance, management and monitoring of water resources and systems in the areas being compared. These, in turn, might arise as the result of culturally (or politically) determined preferences, the initially adopted control paradigms and the associated strategies, and in addition a high level of power distance and uncertainty avoidance [27].

In all the examples where data was collected during the pandemic’s early phases, a combination of factors contributed to some changes in daily domestic patterns of water consumption. In these other studies, we looked particularly for factors that needed to be included in the modified DPSIR framework and for confirmation that factors included in the initial DPSIR framework (Fig. 3) were indeed of some importance. Table 2 summarises the main findings on how various countries have been affected by different phases of the pandemic and makes comments related to the final DPSIR framework (see final sections).

**Table 2.** Context for Istanbul case study. The table lists changes in water use during the pandemic in various countries and comments on factors leading to or contributing to more challenging water management circumstances that might help revision of the initial DPSIR framework

Country	Reported changes in water use during the Covid-19 pandemic in 2020	Main sources of information (academic, trade and media sources all used) and main comments relating to revision of DPSIR framework taking both Covid-19-related and climate/weather influences into account
UK	Decreased demand in Central London Increase in suburban areas Overall raw water use was 25% higher across 10 water companies (effect due to pandemic perhaps 5–10% of total increase; rest due to high summer temperatures) The highest level of demand for 31 years in Thames Valley area; 30-year peak in Severn Trent Thames Water: recoded peak demand levels exceeding 1000 million L/day (over 150 million L/day above norm) The driest and hottest May 2020 on record in England Northumbria Water: 29 L extra/person per day United Utilities 4.6bn extra L of water pumped in early part of pandemic Some supply interruptions may have been partly due to increased domestic water demand in South East England At peak demand, South East Water pumped about 150 million L more than the daily norm	[28–40] Changes in work pattern Increase in water usage with a strong impact of high temperature compounded with Covid-19-related effects Low/lowest reservoir levels in some places could have been challenging if demand had been any higher Partnership working important in managing very high demand Bowsers (portable water tank) and bottled water to deal with interruptions in supply More resilient infrastructure factoring in crises management as well as meeting demand from demographic trends Good public response to appeals to use less water (e.g., demand fell by 30 million L/day in South East Water area immediately after appeal)
USA	Water use increased by up to 21% in some places Delayed morning peak water consumption from 07.00 to 09.00 am Local water crisis driven by extreme weather	[41–43] Increased in domestic water usage Reduced commercial water use Behavioural changes Possible greater use of digital technologies needed
Brazil	Shifted morning water consumption peaks from 08.00 to 10.00 am Decrease in commercial water use by over 30% Increase in residential water use by 11%	[44] Behavioural/social factors Changes in domestic and commercial water usage Changes in morning routines
Türkiye	Unbilled water consumption permitted to help manage the pandemic Higher and lower water demand likely linked to the pandemic and associated temporary migrations	[45,46] Economic disruption for municipalities from falling revenue Changes in water usage (both domestic and commercial) linked to pandemic and higher summer temperatures Low reservoir levels could have been challenging if demand had been any higher
Italy	Complex shifts in water demand Morning peak shifted from 08.00 to 10.00 am	[47] Behavioural and social factors important elements in both stochastic and deterministic aspects of complex shifts in demand
Germany	14.3% higher water consumption Shift in water demand (i.e., later morning peak and higher evening peak)	[48] Increase water usage behaviour changes

#### Reservoir levels

We could only access reservoir data for the UK from readily available data sources. Despite different weather patterns, there is evidence for a decline in reservoir-based water resources in both the UK (Fig. 6 from the London, UK area as an exemplar) and Türkiye (Fig. 4) during the period of the pandemic. Not all of this is necessarily due to it, but perhaps to a combination of lack of ability to replenish reservoirs and additional demands for supply.

Figure 6 shows how Thames Water reservoir levels (%) in London have changed since 1989. As with Istanbul, there are indications in this dataset that the lowest reservoir levels in the London area may occur every 6–7 years. The effect was most pronounced in 1990, with not dissimilar data for 1996/97, 2003, 2011 and 2017/18 as well. However, one difference between the Istanbul and London areas is that London reservoir capacities rarely reach the low levels seen in those that serve Istanbul, perhaps because London has the Thames catchment to call on as a freshwater resource.

## Discussion

The initial hypothesis was that the Covid-19 pandemic would cause more water to be used in Istanbul, a major city that has suffered near drought conditions during the last 20 years. Some increase in water use was seen early in the pandemic and during the summer and early autumn of 2020. However, perhaps due to temporary migrations to other places before lockdown restrictions prevented this, some decline in water use was also recorded. Overall, it seems the maximum additional demand on Istanbul’s water supplies from the pandemic was about 150,000,000 L/day in the summer of 2020. We note that water consumption was even higher in midsummer of 2021. Water consumption may have been higher still in 2020 had it not been for the temporary migrations.

We also wanted to know at the outset whether climate change could make a future pandemic more difficult to manage in terms of Istanbul’s water system. A simple calculation can be done to make a very approximate estimate of what this extra demand could look like. Figure 11 could be interpreted to indicate that as temperatures rise from about 10°C in winter to the summer values of about 25°C, water use per head rises by 20 L/day per person or the equivalent of 300,000,000 L/day for Istanbul as a whole, assuming a constant population. If summer temperatures rose by as much as 8°C (a worst-case scenario) then Istanbul might have to find resources to pump a maximum of 150,000,000 L/day due to higher temperatures and, if another pandemic occurred at such a time when temperatures were very high, another 150,000,000 L/day on top to meet public health needs. Thus, overall, the joint impact of a pandemic and climate change might involve treating and pumping an additional 300,000,000 L/day at peak times. This figure represents about 10% of the average water use in Istanbul at present. This might be a relatively small figure but even this could be challenging if rainfall is lower in the future or if drought conditions coincided with a pandemic and reservoir levels were low at the start of the public health emergency. There are a large number of assumptions and uncertainties in such figures and outlooks. These include assumptions about a stable population in Istanbul, no further growth in the water use per head, little change in infrastructure and the difficulties in projecting climate into the future via scenarios and readily available down-scaling tools. There are also uncertainties around the capacity of the water system to cope with the changed patterns of use that accompany a pandemic.

The international comparisons tend to support the main findings of this study. For example, in the UK, the overall increase in water use due to the pandemic was thought to be between 5% and 10%. By combining information from a range of sources we can see that more than one water company was pumping exceptional levels of water to consumers, of the order of an additional 150,000,000 L/day in these cases. Superficially this is strikingly similar to the figures for Istanbul but in at least one of these UK examples, South East Water, this was to a much smaller population than Istanbul’s. In Germany and Brazil, water use also increased by similar amounts.

We suspected at the start of this study that in such a unique situation as the Covid-19 pandemic many strategic and more topical issues would emerge. This is why we took the DPSIR approach as such strategic and emergent factors might be important for managing water resources in the future. We were surprised at the number of these we found. We discuss these below.

### Strategic and emergent issues and factors

Although only limited comparison can be made between regions, countries and local areas as the Covid-19 pandemic is still playing out in many places, some strategic and emergent issues can be identified that might be relevant to improving the future resilience and adaptability of water systems facing hazards such as pandemics and climate change. When trying to figure out what the strategic and emerging issues are, it is important to consider a wide range of socio-ecological, socio-economic, socio-technical and governance issues. Differences between countries and local areas in their approaches to hazards, risks and emergencies, as well as their capacities and approaches to adaptation in different but related environmental and epidemiological situations, must be taken into account (see [27] for insightful discussion of these matters). By the way of example, if Türkiye and the UK are compared then it can be argued that similar impacts occurred on water systems despite differences in strategic approaches (say, with respect to governance of water supply). As more information emerges about water use and water resources in the pandemic period a more complete set of strategic and emergent issues will emerge.

### Strategic issues

Thus, in the UK, by May 2020, the pandemic was already impacting many areas of life because of public health restrictions. As impacts on the water system were already being recognised at a high level, Water UK (representing the UK water companies) and Ofwat (the water regulator) decided to work collaboratively and an early report [49] found many actual and potential impacts on water companies, including unpaid bills and other consequences of raised unemployment rates, lower company income due to tariffs reductions for those many customers with reduced levels of activity, more household consumption, and less non-household consumption. Similar issues arose in Türkiye even though, below the national level, responses were less strategic in some senses with individual municipalities taking different approaches to, for example, whether or not to bill customers for using utility services for a three-month period from March 2020 when employment and thus earnings were affected by public health restrictions (e.g., [45,46]). This led to unbilled consumption and lower revenue for the municipalities. It also inadvertently complicated understanding of patterns of water consumption in some places in Türkiye in a way that has yet to be fully resolved (see also Table 2 for key information in relation to several countries).

Despite the importance of water in all aspects of life and its additional importance during almost all serious disease outbreaks, it is noteworthy that despite the level of concern about the impact of Covid-19 on water systems, a review of governmental responses in 27 European countries [50] revealed that Covid-19 pandemic policy measures were focused around economic ones and water had only limited interventions. Those that did exist in some countries predominantly consisted of short-term measures to ensure uninterrupted water supply and to cushion the impact of the loss of income. The findings in this study suggest a more complete view of the water system in managing emergencies with public health and environmental elements is needed.

Concerns about the impact of Covid-19 on water consumption in many other parts of the world are emerging. For instance, Sivakumar [51] argues that efforts to control the spread of Covid-19 will likely increase the water demand and worsen water quality, leading to additional water planning and management challenges and an urgent need for issues to be addressed. For Sub-Saharan Africa (SSA), Anim and Ofori-Asenso [52] suggest that a wide range of different approaches were thought necessary. These were made more important by rapid population growth and the impact of climate change which would increase drought risk. Solutions might include: (i) increasing the efficiency of water use by implementing strategies for conservation of available resources (ii) nature-based solutions to help with water storage and supply. An approach based around ecosystem services and community engagement has worked in the case of New York, USA [41,53–55].

### Emergent water issues

In the course of the study a number of emergent issues were uncovered ranging across social, economic and environmental fields. Most of these emergent issues arise as part of an ongoing comparison of Türkiye and other countries. Most of the emergent issues arise from Türkiye and the UK but some are from other countries and regions.

#### Water quality

As evidenced in Fig. 10, a lot of water has been used for disinfecting public spaces. Many kinds of cleaning, disinfecting and bleaching agents were probably used during the pandemic, some of which may have been used in quantities and amounts that were not expected when the national and international risk assessments were done for these materials. When released into the environment, in this case through surface water drains or directly onto soil near the areas disinfected, bleach and other chemicals can release chlorine that reacts with organic matter in the soil, water and air to form a range of compounds, some of which might be classed as halogenated compounds subject to restriction. Other materials, such as detergents, can have toxic effects on aquatic systems if they enter these systems at high enough concentrations. Thus, it is possible that such practices could have environmental impacts that have not yet been quantified as these compounds could be toxic to wildlife, carcinogenic or mutagenic, accumulate in the food chain and eventually impact humans. Not all water used for disinfection passes through a treatment facility before entering the natural environment. Thus, the products used for hygiene purposes during Covid-19 times may end up in rivers and in the sea.

Additionally, the disinfection products may also infiltrate the soil and impact land, plants and animals. As the purification process used by most water treatment plants is achieved by bacterial action, the introduction of chemicals in high concentrations (such as bleaches or disinfectants) could significantly impact treatment plants. More evidence is needed on these emergent issues linked to public health measures taken in the public realm of many urban areas.

#### Increased sewage system blockages

This was an issue for the UK and probably other countries as well. As people stayed home more, increased flushing of inappropriate articles through the domestic toilet systems caused blockages in the sewerage systems [56–58]. Popular news outlets carried dramatic images of the increased blockages occurring as early as April 2020. It seemed that Covid-19 lockdowns were followed by a rise in blockages [59]. Media messages to only allow permitted materials into the sewage system accompanied such images.

#### Economic impacts on water companies

Increased costs of sewer blockages (costing about £3M) were only one of the economic impacts of the pandemic on Thames Water, UK. As people lost their jobs and experienced financial difficulties, they were advised to contact their water companies for help with deferring their water and sewerage payments. This led to bills going unpaid. There were also increased costs of running water infrastructure as a result of changes in the pattern and size of the demand. Thames Water posted a pre-tax loss of £246.5M in its interim report for 2020/21 [60]. Many of the factors involved in this loss involved social/economic ones (help to less well-off domestic customers; loss of revenue from business customers during the pandemic; reassessment of risks partly to account for the pandemic’s wider implications) as well as other cost issues linked to supply, sewage and infrastructure. Parts of this loss, such as the increase in bad debt, were attributed directly to the pandemic. Similarly, Southern Water [61] refers to several impacts of the Covid-19 pandemic, including a 7% rise in water use from 127 to 136 L/person per day; leakage up by about 4%; increased operating costs of £2.7M together with redistribution in income from different customer sectors; help for vulnerable customers; and a need for re-analysis of a range of business risks. The full effects of the pandemic on the water sector may not be known for some time.

In Ankara, capital of Türkiye, during lockdowns, real-time usages were not billed due to governmental statements and restrictions (to protect workers and public health) and, consequently, municipalities’ revenues were disrupted, which might be even worse in a further pandemic or under currently unexpected pressures, such as those that might arise under climate change [62].

#### A wider view of water and water efficiency

The events of the pandemic also suggested that people may need to take a wider view of water resources and act accordingly. For example, in order to act on advice to consume less water, they may need to reuse water used while cooking for watering plants, use less water when washing and showering and, if possible, plant drought-resistant seeds in gardens and parks [63].

#### Global issues

By disrupting economies and causing millions of deaths globally, the Covid-19 pandemic has likely had a serious impact on progress toward the Sustainable Development Goals (SDGs) and further compromised the 2030 targets. Some argue it is important to introduce further cost-effective and innovative policies for achieving those SDGs [64]. More generally, there were concerns about preventing water contamination as the virus can survive up to several days, perhaps longer in low temperature regions. Even if sewage treatment methods and approaches such as chlorination and ultraviolet (UV) irradiation have the ability to eradicate Covid-19 in the water, the possibility of contamination may be high in areas where sewage is untreated [65]. Interestingly, water systems can also be part of the early warning systems for Covid-19 (or other types of pandemics or diseases). In many parts of the world, this form of monitoring for Covid-19 has been initiated (e.g., in the Netherlands) [66].

Emergencies, such as the Covid-19 pandemic [67], can compound the pressure on water usage and availability that are already under pressure from climate change in some parts of the world.

#### Local climate

A similarity between Istanbul and the London/South East England area is that both regions are likely to experience higher temperatures and more periods of dry weather under climate change scenarios [68–70]. For some parts of the UK, there are substantial concerns about water resource and supply issues due to climate change alone, especially after 2050. Potential supply shortages of between 1220 and 3900 million L/day are possible for the UK – that is, a supply equivalent to that for between 8.3 and 19.7 million people [71]. Actions are in hand to address the climate issues [39,40] but issues linked to the pandemic may need to feature more explicitly than they do at present. Economic growth in South East England together with rapid demographic changes, driven in part by housing-led regeneration, are adding to water quantity and quality issues with a pause on housing development in place during 2022 that will have long-term impacts on a range of social issues.

#### Water resources – reservoirs

For a range of reasons reservoir levels in various countries were at low levels during the pandemic; this was certainly so in parts of Türkiye and parts of the UK as this study indicates. We have not examined aquifer data as that is not a major factor relevant to the main case study area of Istanbul. Additional consideration of the operational margin needed to maintain a viable live capacity may be necessary given pandemic experiences and upcoming climate change impacts on precipitation patterns. New reservoirs are proposed for South East England [39,40] that allow for climate change, but perhaps not a future pandemic whose control measures extend into hot weather periods when demand for water increases, especially if this occurred in a period of drought.

#### Reducing risks with better data flows and analysis

The substantial changes in raw water use during the pandemic and the pressure on such compound the risks to water systems presented by climate change and other socio-economic events such as the current pandemic, and suggest that much greater use of digital technologies may be necessary for use in a reimagined water infrastructure system [41].

## Conclusions

In this paper, we have shown how a range of factors relevant to water supply and resources could be affected by the Covid-19 pandemic in several different locations, principally in Istanbul. Table 3 summarises from this evidence how the pandemic had impacts on the water system of Istanbul, the wider London area and other countries and localities. Full lockdowns have major impacts as many people, except key workers, have to stay at home and so use more water for domestic and hygiene purposes and perhaps also in gardens and other leisure uses (e.g., children’s paddling pools and hoses in hot weather). The impacts of this public health emergency on the water systems of Istanbul, South East England and other countries appear to be similar. Istanbul may have been saved from supply issues by the temporary migration of people away from this centre of population.

**Table 3.** Classification of different factors related to the pandemic and weather under the influence of climate change and their possible impacts on water dynamics and the water environment

Factors	Impacts
Promoting hygiene	Cause additional pressure on water resources, increase water usage
Street disinfections	Increase water usage, pressure on sewage system, environmental pollution
Staying home	Change water consumption trends, increase morning water usage, pressure on sewage system
Working remotely	Increase water usage, and changes in the balance of domestic and commercial usage
Population movement	Increase or decrease water usage, changes in regional usage
Full lockdowns	Heavily increase water usage, more pressure on sewage system, blockages, water shortages, decrease water reservoir levels

**Figure 16:**
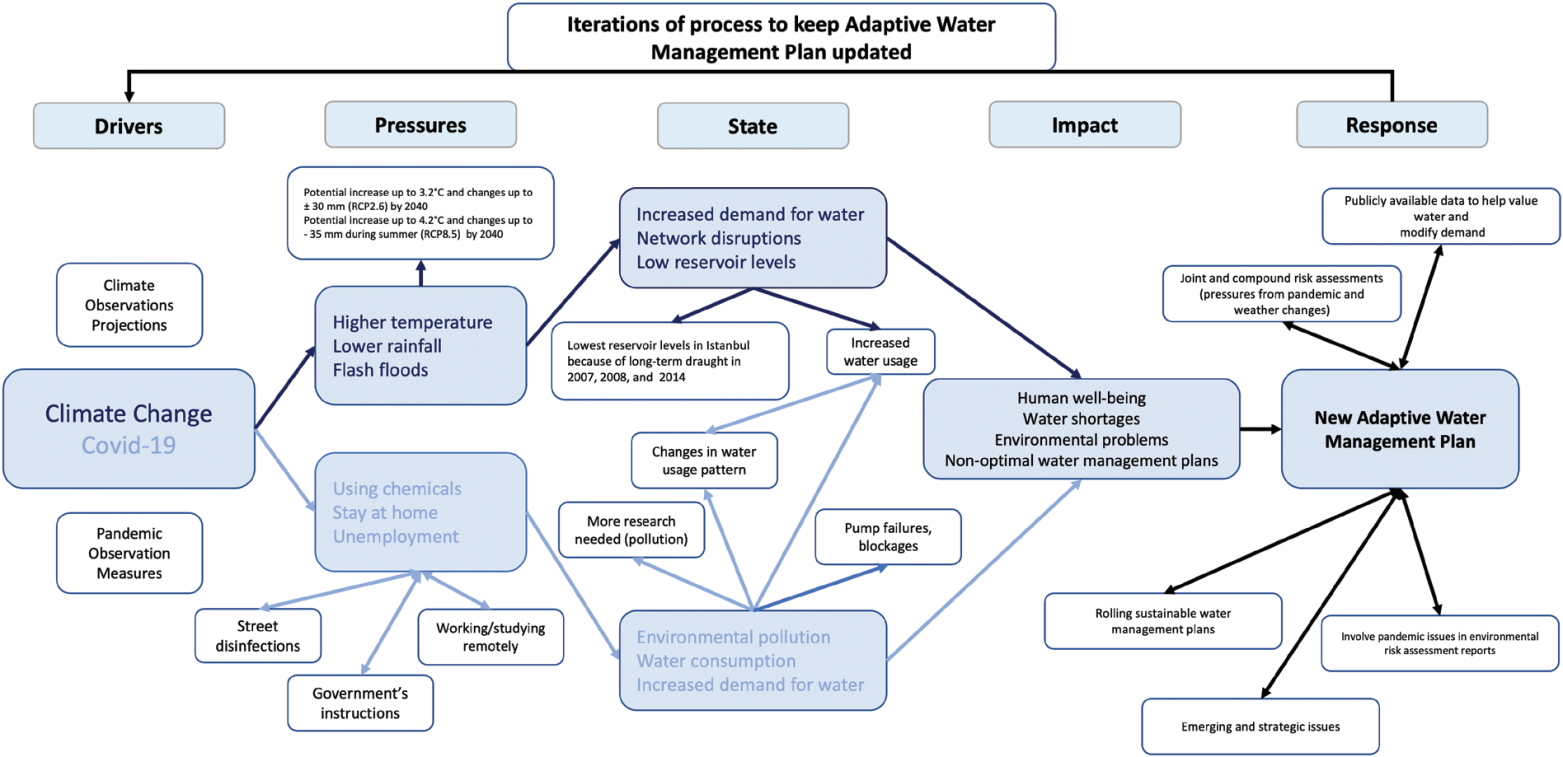
Enhanced DPSIR framework.

We have taken the summary findings from our study of the Covid-19 period in Istanbul and elsewhere and used these so that Fig. 3 has been modified from an initial to an enhanced DPSIR framework to set out at the next level of detail the factors that may be affected by both climate change and the pandemic. This diagram perhaps relates most closely to Istanbul but may also have relevance to other places, such as the London area and other large conurbations. The modified diagram, Fig. 16, might help in thinking about new adaptive management plans where there will be a need to provide publicly available data, assess actual and potential issues with affected communities and take account of both drivers of long-term trends and shorter-term extreme events (such as pandemics or droughts). We hope a DPSIR framework may help major municipalities and their hinterlands develop such ways of managing water resources and supply in the future. This study suggests that the factors involved may be the same for different localities but need to be given different weights to account for differing environmental, social and economic circumstances.

## Data Availability

The datasets generated during and/or analysed during the current study are available in the repository: https://doi.org/10.5522/04/19122179.

## Note

1 On 31 December 2019, a novel coronavirus was identified in Wuhan, China. Globalisation facilitated the rapid spread of this Covid-19 virus. The first confirmed case outside of China was recorded in Thailand on 13 January 2020. Alarming levels of spreading and severity obliged the World Health Organization (WHO) to characterise Covid-19 as a pandemic. As of 28 November 2022, there have been around 637 million confirmed cases of Covid-19, and around 6.6 million deaths globally. Daily cases are still around 200,000, and around 13 billion vaccine doses have been administered (see [67]).
